# Barriers and facilitators to implementing a continuing medical education intervention in a primary health care setting

**DOI:** 10.1186/s12913-022-08019-w

**Published:** 2022-05-13

**Authors:** Teresa Reis, Inês Faria, Helena Serra, Miguel Xavier

**Affiliations:** 1 Nova Medical School, Comprehensive Health Research Centre (CHRC), Universidade NOVA de Lisboa, Campo Mártires da Pátria, 130, 1169-056 Lisbon, Portugal; 2 Research Centre in Economic and Organizational Sociology, Lisbon, School of Economics and Management, University of Lisbon (CSG-SOCIUS/ISEG, U.Lisboa), Lisbon, Portugal; 3 Interdisciplinary Centre of Social Sciences (CICS.NOVA), NOVA School of Social Sciences and Humanities, Universidade NOVA de Lisboa, Lisbon, Portugal; 4 National coordinator of mental health policies, Portuguese Ministry of Health, Comprehensive Health Research Centre (CHRC), NOVA Medical School, Universidade NOVA de Lisboa, Lisbon, Portugal

**Keywords:** General Practitioners, Primary health care, Continuing medical education, Barriers, Facilitators, Mixed methodology

## Abstract

**Background:**

Continuing medical education (CME), as a systematic attempt to facilitate change in General Practitioners’ (GPs) practices, is considered crucial, assuming that if physicians are up-to-date, they will change and improve their practice, resulting in better performance and ultimately better patient care. However, studies continue to demonstrate considerable gaps between the real and ideal performance and patient-related outcomes. The objective of this study was to explore GP’s perception of the factors affecting the implementation of a CME digital platform in a primary health care setting in Portugal.

**Methods:**

Our work is framed in a larger effectiveness-implementation hybrid type 1 study, where a Digital Behaviour Change Intervention (DBCI), called ePrimaPrescribe, was developed and implemented with the aim of changing benzodiazepines (BZD) prescribing patterns. Our design used mixed methodologies to obtain an enriched knowledge on GPs’ perspectives on the facilitators and barriers to implementing a Digital Behaviour Change Intervention (DBCI) applied to CME. To do so, we used data coming from an onsite questionnaire, an adapted version of the Barriers and Facilitators Assessment Instrument (BaFAI) and in-depth interviews.

**Results:**

From the 47 GPs successfully included in the intervention arm of our cluster-randomized effectiveness study, we collected 37 onsite questionnaires, 24 BaFAIs, and performed 12 in-depth interviews. GPs reported as the main barriers to CME a lack of time, a perception of work overload, a lack of digital competence, a lack of digital infrastructure, and motivational and emotional factors. They reported as facilitators to CME delivered through a DBCI the convenience of the delivery method, the practical and pragmatic characteristics of the content, and the possibility for CME to be mandatory.

**Conclusions:**

The perceptions of the barriers and facilitators reported by GPs represent an important contribution to improving knowledge regarding the factors influencing the implementation of CME in primary health care settings. We consider that our study might bring useful insights to other countries where primary health care plays a central role in the provision of care.

**Trial registration:**

ClinicalTrials.gov number NCT04925596.

**Supplementary Information:**

The online version contains supplementary material available at 10.1186/s12913-022-08019-w.

## Background

Continuing medical education (CME), defined as any and all ways through which doctors learn after the formal completion of their training [1], is a systematic attempt to facilitate change in physicians’ practices [2]. Efforts have been made to promote physicians’ participation in CME, assuming that if they are up-to-date, they will change and improve their practice, resulting in better performance and ultimately better patient care. A meta-analysis aimed at determining the effect size of CME interventions on physician knowledge, physician performance and patients outcomes found that the largest effect sizes were found with multifaceted educational programs, longitudinal workshops, interactive small groups, and case discussion interventions, delivered to single discipline participant types [3]. However, studies continue to demonstrate considerable gaps between the real and ideal performance and patient outcomes [4]. This gap between the real and ideal performance in the health care system increases uncertainty about the role of CME, particularly in terms of changing behaviours associated with everyday clinical practice such as prescribing. Given this gap, there might be a need to better acknowledge the facilitators and barriers to the implementation process of CME interventions.

In recent years, there has been an exponential growth in the potential of e-health CME interventions. E-learning approaches have shown similar effects on outcomes as conventional face-to-face approaches, in terms of learners’ knowledge and learner’ satisfaction, and similar limitations in terms of changes in processes and outcomes of care [5–8]. There are some benefits to an e-learning approach to educational materials: educational content can be easily updated, it can be provided to meet individual learning needs, and it can be delivered at any time and in any place, depending on the technology used [5, 7–9]. An increasing number and variety of CME e-learning opportunities are now available for primary healthcare, considering that in this setting interventions with a lower demand on professional time and limited budget are particularly interesting.

Digital Behaviour Change Interventions (DBCIs) are behaviour change interventions that involve computer technology or digital encoding of information [10]. This specific type of behaviour change intervention has a particular potential for its successful application to CME interventions in primary health care settings, given its low unit-cost, high reach, and its effective and acceptable ways of benefitting individuals and society. Compared with human-delivered interventions, DBCIs usually deliver the intervention content with a high degree of fidelity. They may improve health by improving health professional adherence to evidence-based guidelines and enhancing health professional effectiveness or efficiency [10]. Despite the fact that there is still a gap in the literature regarding health professional’s perspective over CME interventions after the COVID pandemic, before this period, online CME was already considered appealing since clinicians valued the possibility of learning when they had time, at their own pace, and at a lower cost [7]. We could infer that clinicians’ acceptance and availability after a period when most had to adjust to using more learning online resources would increase. This might mean a disruptive innovative change to preferred learning methods, with the very high participation in first-time online conferences being a clear example of this shift.

In Portugal, General Practitioners (GPs) have a four-year mandatory residency training period. During this time, knowledge acquisition in different specialized areas, such as cardiology, paediatrics or psychiatry occur in hospital settings for a few months. After completing their residency, and when choosing to join primary health care units integrated in the Portuguese National Health Service (NHS), GPs are granted with 15 days a year to engage in voluntary CME training, such as conferences and symposiums. Moreover, in most units there is a voluntary and non-accredited training program, with weekly to monthly presentation and discussion sessions, that are either held as traditional discussion meetings or involve invited participating specialists. There is no need for recertification after residency completion, hence compliance with CME is affected by personal, motivational and organizational factors.

Our work is framed in a larger effectiveness-implementation hybrid type 1 study, where a DBCI called ePrimaPrescribe was developed and implemented, with the aim of changing benzodiazepines (BZD) prescribing patterns and, in a broader sense, the management of mental health issues in a primary health care setting. The objective of the analysis presented in this paper was to explore the perception over the factors that affected GPs’ voluntary compliance with our CME digital platform in primary health care settings. Identification of the barriers and facilitators to CME compliance is important for both CME providers and GPs since understanding the facilitators and barriers to implementing learning from CME can have important implications for the effectiveness of health care delivery. Hence, with this work we hope to contribute to shaping of CME, adapted to the characteristics and challenges of real-world routine clinical practice in primary health care settings.

## Methods

### Design and setting

Our study used a mixed-methods sequential design to obtain rich knowledge on GPs’ perspectives on the facilitators and barriers to implementing a Digital Behaviour Change Intervention (DBCI) applied to CME. The primary intention of this design was to add a qualitative strand to in-depth initial quantitative results [11].

The quantitative approach allowed us to obtain measurable evidence on providers’ perspectives, while the qualitative approach provided a deeper understanding of their perceptions.

We started by collecting data concerning the barriers and facilitators to implementing a DBCI using two questionnaires. The questionnaires allowed us to explore some of the practices, perspectives and points of view of a large number of individuals, though the depth of the research was limited by the standardized approach, which failed to account for the contextual and procedural circumstances and dynamics. This resulted in a lack of sensitivity to explore the differences, inconsistencies, meanings and arguments that emerged [12]. In order to empirically capture the current trajectories and perceptions of the implementation of a DBCI applied to CME in the primary health care settings, it was necessary to apply qualitative methods of data collection. Through semi-structured interviews, the analytical focus centred on narratives about GPs’ continuing educational experiences using online resources, whose trajectories assume particularities that are not likely to be captured through an overly structured script.

The setting for our intervention were primary health care units in a rural region in Portugal, with an area of 7.393 km^2^, an estimated population of 166.706 inhabitants (2011), a population density of 22.5 inhabitants/km^2^. All primary health care units from the Central Alentejo region were considered eligible. We contacted each primary health care unit coordinator, explained the project, and invited their participation. Of the total 250 GPs working in these primary health care units, 110 were included in our study. The 110 GPs included had similar characteristics regarding type of primary health care unit where they were prescribing (UCSP vs. USF), their sex, age, years of clinical experience and training in mental health, hence they were considered representative of all target GPs. Portugal has an NHS with free coverage for the entire territory. Primary health care is responsible for the provision of care and has a gatekeeping function for hospital care. In Portugal, the NHS distinguishes two types of primary care units. The default one is the ‘personalized care unit’ model (UCSP), in which professionals receive a fixed salary; the second is the ‘family health unit’ model (USF), which enjoys higher functional and organizational autonomy [13], and where GPs might have a mixed payment scheme that includes salary, capitation, and pay for performance [14].

### Onsite questionnaire and BaFAI questionnaire

#### Sample and data collection

We distributed, at baseline and at a final implementation onsite visit, a questionnaire containing three multiple answer questions and three short answer questions concerning motivations, expectations and barriers to utilization of our DBCI (Additional file 1). At the final implementation onsite visit, which occurred 12 months after intervention implementation, we distributed the Barriers and Facilitators Assessment Instrument (BaFAI) [15] to the participating GPs included in the intervention arm of our effectiveness trial. The BaFAI included a set of 25 issues related to barriers to the implementation of innovations, organized into four categories of barriers: barriers deriving from the characteristics of the practice/innovation; barriers deriving from the characteristics of the professionals; barriers due to patient characteristics; and barriers arising from the intervention context [15]. This questionnaire was delivered only to participants who actually used the DBCI online platform. We characterized participant GPs according to their utilization of the DBCI, the type of primary health care unit where they were prescribing (UCSP vs. USF), their sex, age, years of clinical experience and training in mental health.

#### Data analysis

We performed descriptive statistical analysis to describe GPs’ socio-demographic characteristics, perceived motivations, expectations for using the DBCI, and the main barriers arising from the implementation process into their daily clinical activities.

### Semi-structured interviews

#### Sample and data collection

We conducted semi-structured interviews with GPs included in the intervention arm of our effectiveness study. Questions integrated in the interview’s script were based on several sources: on a previous literature review; on the suggestions of the expert who evaluated the intervention before implementation; on information collected from the BaFAI questionnaire; on information from the short-answer questions from the onsite questionnaire; and on topics mentioned in exploratory group discussions held at primary health care units included in the intervention arm of our effectiveness cluster randomized trial. Using a similar language and nomenclature to these instruments, the interviews served to understand the perceptions concerning implementation of our DBCI. The semi-structured in-depth interviews allowed us to reconstruct and deepen procedural aspects, relating them to the experiences and rationalities around the major dimensions of analysis.

One of the main topics of the interview concerned the barriers and facilitators to implementing a DBCI as a delivery method for CME in primary health care settings. Here, a barrier was defined as any factor that was perceived as limiting or restricting GPs’ compliance to CME. We focused on barriers that could be changed by an intervention. As a result, we did not consider sex or ethnic background as barriers. Accordingly, we defined facilitator as any factor promoting compliance to CME.

We conducted a pre-test interview in order to: validate the content of the data collection instrument; to check familiar and lexical options; stabilize the categories and identify more problematic, or emerging ambiguous interpretation issues; discover potential errors; and to assess the duration of the interviews.

We defined an initial convenience subject sample for our interview balanced for the same characteristics considered for the onsite questionnaire and BaFAI. These criteria were chosen considering their probable influence on behaviour towards CME.

#### Data analysis

Each interview was transcribed and analysed by two researchers. Interviews were coded, synthesised, and categorised according to similarities of meaning. Patterns within and across categories were analysed and grouped into themes. Categories and themes were driven by literature concerning barriers and facilitators to CME. Coding continued until no new concepts emerged from the data. Coding, category-building procedures, and thematic analysis were discussed by the authors until consensus was reached. For instance, there were some doubts in the process of categorization, particularly regarding the inclusion of ambiguous statements in more than one category, or sub-category. It was in the discussion process that we considered that this ambivalence of perspectives towards digital CME from the part of GPs was in itself a significant finding of the study. The qualitative analytical framework used consisted of a content and discourse analysis [16, 17] made with the support of the software Atlas.ti [18]. The initial categories for the qualitative data analysis were defined based on a preliminary literature review and descriptive analysis of the onsite questionnaire and BaFAI, to which specific categories stemming from the interviews themselves were added. This process was developed in collaboration in order to refine and discuss the kind of categorization created and its conformity with the in loco experience of the empirical research.

Data were converted into segments of relevant information and concepts, then organized into categories, and the results were analysed and interpreted. Quotes were chosen to illustrate the topics, meanings and contexts provided by the interviewees. To maintain participants’ confidentiality, the names of the interviewees and of other providers/institutions have been removed from the transcripts. The interviewees are identified in the text by their sex, years of clinical practice, previous training in mental health, and the type of primary health care unit in which they prescribe.

### Ethical considerations

Participation was voluntary and informed consent was obtained from all enrolled participants. All information was handled with confidentiality. Each interview was given an anonymized coding number. This study was approved by the Ethics Committees of the Regional Health Administrations of the region where the study was implemented.

## Results

### Participants

**Fig. 1:**
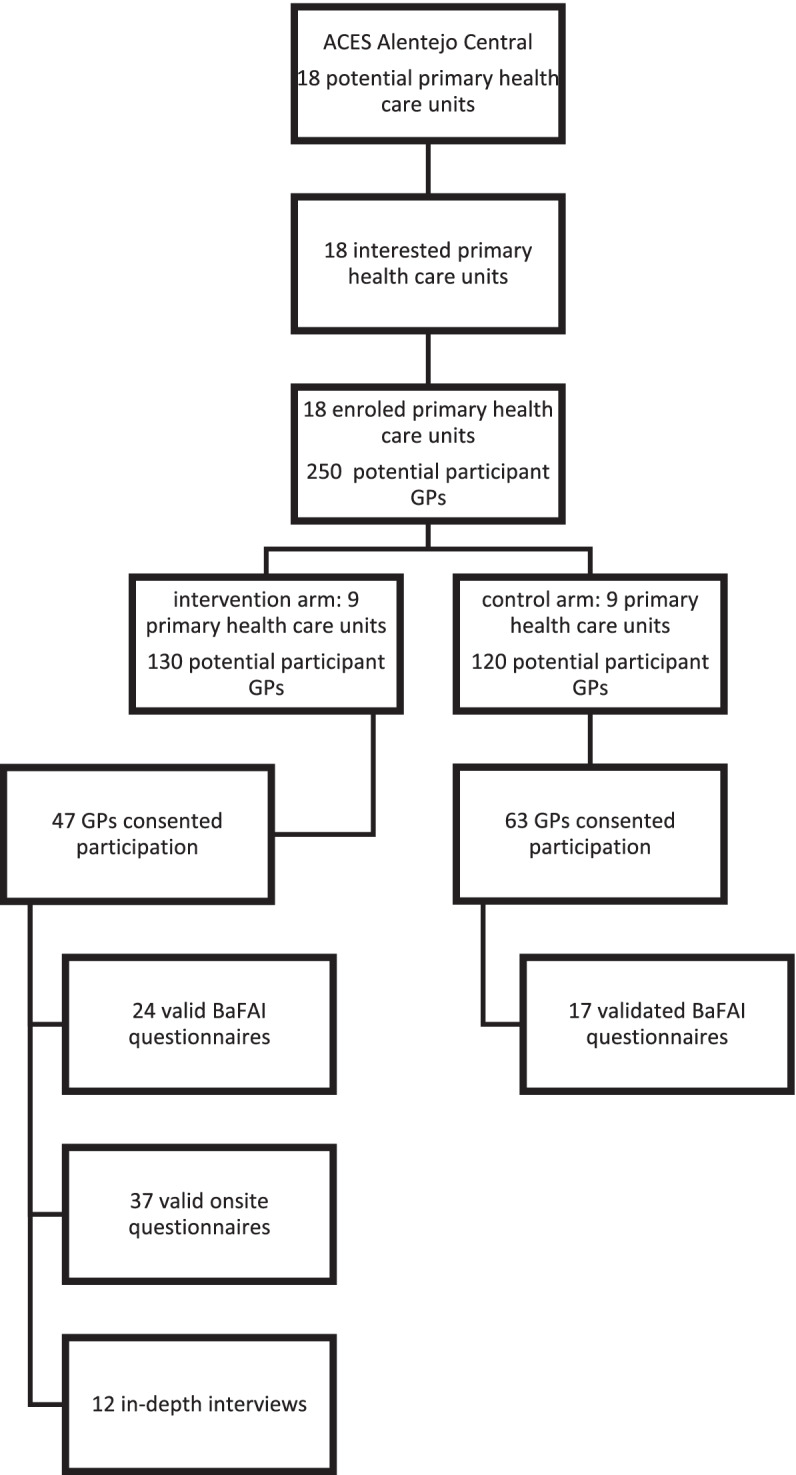
Distribution of study participants and distribution of data collection

During a twelve-month period, 47 GPs were successfully included in the intervention arm of our cluster-randomized effectiveness study, and had access to our ePrimaPrescribe DBCI platform (Fig. 1). We collected data from onsite questionnaires that were completed by 37 intervened GPs (Fig. 1). Sixty-eight percent of respondents to this questionnaire had used the DBCI platform and 72% were prescribers at USF primary health care units. The respondents’ mean age was 47 years old. The mean years of clinical experience was 20 and 57% of GPs had at least some specific training in mental health (Additional file 2).

The BaFAI was completed by 24 GPs included in the intervention arm, and by 17 GPs included in the control arm of our cluster-randomized effectiveness trial (Fig. 1). We found that most respondents were prescribers at USF type primary health care units; that the mean age was 46 years old; the years of clinical experience varied from 4 to 18; and that there was a balanced distribution of responders with and without training in mental health (Additional file 3).

Regarding the in-depth interview, GPs were selected from the nine primary health care units included in the intervention arm of our effectiveness cluster-randomized trial. We defined a convenience sample for participating in the in-depth interview balanced for the following selection criteria: working in personalized health care units (UCSP) or in family health care units (USF); having or not specific training in mental health; having used or not the ePrimaPrescribe platform during the implementation phase of the study. These criteria were chosen considering organizational structure, policies and routines influence on behaviour towards implementation of the DBCI online programs [19], and specifically of the ePrimaPrescribe program. We carried out 12 interviews until saturation of the themes and topics found was reached, with no further new reported information (Fig. 1). Of the total interview participants, 58% were prescribing in USF type primary health care units. Fifty-eight per cent of respondents were male and 58% had effectively used the platform. The mean age was 54 years-old and the mean number of years of clinical practice was 27. Our sample was balanced for previous training in mental health (Additional file 4).

We chose to report the perspectives that were more significant and consistent across the different data sources.

### Motivation and expectations

There was a high degree of motivation to participate in the study when it began, which decreased by the end of the study. The majority of respondents had actively used the DBCI platform, and they considered its implementation feasible, both at the beginning and at the end of the study. At the beginning of the study, the participants considered that the platform would have an impact on changing their BZD prescribing patterns, but the number of GPs reporting this impact significantly decreased by the end of the study (Table 1).

**Table 1:** Results from the onsite questionnaire multiple answer questions

	t0	t12	Fisher exact test
Degree of motivation to participate in this study			
a) None	0 (0%)	0 (0%)	*p* = 0,614
b) Little	0 (0%)	8 (32%)	
c) Some	22 (59,46%)	14 (56%)	
d) A lot	15 (40,54%)	3 (12%)	
Do you think it is feasible to implement online training programs like ePrimaPrescribe?			*p* = 0.121
a) yes	35 (94,59%)	29 (87,88%)	
b) no	2 (5,41%)	4 (12,12%)	
Do you consider that the use of the ePrimaPrescribe platform will have an impact on changing your benzodiazepine prescription pattern?			*p* = 0.138
a) yes	36 (100%)	16 (69,57%)	
b) no	0 (0%)	7 (30,43%)	

When asking about the main motivations and expectations regarding compliance with the implemented DBCI, two themes were mentioned more frequently: knowledge acquisition and practice improvement. Some participants reported that the DBCI made them more aware of the need to make a deep reflection about the consequences of their clinical behaviour, in this case, specifically their prescribing patterns.

In the following section, an analysis of findings about barriers and facilitators will be presented, and an example of a direct quote to demonstrate the conclusions of the analysis will be included.

### Barriers

#### Time

Both at baseline and at the end of intervention implementation, the lack of time was mentioned as one of the most important factors limiting CME implementation, regardless of it being online or onsite (Table 2). In fact, implementation of the online format was mentioned as being more significantly affected by the time factor, since its utilization had to occur outside of office hours. Despite most GPs having 15 days a year granted for CME, most mentioned that this time was not actually available to be used, due to work overload and the difficulty of rescheduling the excessive number of patients and appointments that would accumulate after a period of absence to comply with CME.

"the excess work we have, I can't afford the luxury of taking off the fifteen days of training we're entitled to a year, isn't it, for, for the free service commission, I can't take them off, because I can't, simply. I don't have anywhere to refer my patients appointments [if I’m on a leave]” (Interviewee 2, female, 39 years old, 9 years of clinical practice, with mental health training, prescribing at an UCSP primary health care unit, used the ePrimaPrescribe DBCI platform).

**Table 2:** Results of the BaFAI

Factors identified as barriers	Intervention (*n* = 24) (% of total)	Control (*n* = 17) ((% of total)
Characteristics of the practice/innovation		
The online platform leaves enough room for me to make my own conclusions—compatibility	1 (4%)	1 (6%)
The online platform leaves enough room to consider my patients related mental health needs – specificity	0 (0%)	0 (0%)
The online platform is a good starting point for my self- study – didactic flexibility/benefit	0 (0%)	1 (6%)
Working according to the online platform is too time consuming – time investment	13 (54%)	10 (59%)
The online platform does not fit into my ways of working at my practice—attractiveness	7 (29%)	6 (35%)
The lay-out of this online platform makes it handy for use—attractiveness	2 (8%)	4 (23,5%)
Barriers deriving from the characteristics of the professionals		
I did not thoroughly use nor remember the online platform – training attitude	10 (42%)	10 (59%)
I wish to know more about the online platform before I decide to apply it—innovation doubts	8 (33%)	10 (59%)
I have problems changing my old routines. motivation and role	10 (42%)	10 (59%)
I think parts of the online platform are incorrect perception knowledge	0 (0%)	0 (0%)
I have a general resistance to working according to protocols participation	3 (12,5%)	3 (18%)
Fellow doctors (GPs) do not cooperate in applying the online platform—involvement	3 (12,5%)	7 (41%)
Other doctors or assistants do not cooperate in applying the online platform—involvement	2 (8%)	4 (23,5%)
Primary health care coordinators do not cooperate in applying the online platform—involvement	3 (12,5%)	4(23,5%)
It is difficult to apply the ePrimaPrescribe platform … because I am not trained to use online platforms – work style	2 (8%)	2 (12%)
Barriers due to patient characteristics		
Patients do not cooperate in applying the online platform readiness to change	8 (8%)	3 (18%)
It is difficult to apply the ePrimaPrescribe platform to patients due to ethnicity—characteristics	4 (17%)	4 (23,5%)
It is difficult to apply the ePrimaPrescribe platform to patients of low socio-economic background—financial situation	5 (21%)	2 (12%)
It is difficult to apply the ePrimaPrescribe platform to older patients (> 65 years old) age	6 (25%)	3 (18%)
It is difficult to apply the ePrimaPrescribe platform to patients that rarely come to the primary health care unit—number of patient contacts	15 (62,5%)	6 (35%)
Barriers arising from the intervention context		
Working according to this online platform requires financial compensation	1 (4%)	1 (6%)
It is difficult to apply the ePrimaPrescribe platform if there is not enough supportive staff—support staff	3 (12,5%)	4 (23,5%)
It is difficult to apply the ePrimaPrescribe platform if the instruments needed are not available—equipment suitable for practice	17 (71%)	13 (76%)
It is difficult to apply the ePrimaPrescribe platform if physical spaces are missing (eg consultation office with computer)—location of facilities	16 (67%)	9 (53%)
It is difficult to apply the ePrimaPrescribe platform if physical space is lacking (e.g. consulting room)- location of facilities	7 (29%)	9 (53%)
${x}^{2}$ Pearson = 162,15 Pr = 0.130 Fisher’s exact = 0.012		

Work overload, which led GPs to attempt to do many things at the same time because of the limited time they had to fulfil the different tasks they are expected to perform, was referred as anxiogenic. GPs expressing this perception ended up not even trying to use the DBCI, thus demonstrating how the lack of time and its effect on participants’ emotions directly influenced intervention implementation.

#### Work overload

As a barrier to CME implementation, GPs mentioned feeling overwhelmed by their need to receive training in too many different areas, since they are first in line as primary medical care physicians and patients come with very different complaints. Moreover, they also mentioned the fact that they face excessive bureaucracy in their role, leaving little time for training or further studying. In some ways, the GPs’ underlying GPs discourse translates into frustration over the impossibility of “doing it all”.

"The fields of Family Medicine are many, many, and, and therefore it is difficult to maintain continuous training in, in cardiology, in diabetes, in child health, in, 'you see, in mental health, in, it's this, that, the other. So I think, hmm, they're a lot, they move in many areas at the same time, don't they?” (Interviewee 5, male, 67 years old, 40 years of clinical practice, with mental health training, prescribing at an USF primary health care unit, used the ePrimaPrescribe DBCI platform).

#### Digital competence

Barriers related to skills were also mentioned, such as lack of digital know-how because of age. Senior GPs found digital tools challenging due to a lack of knowledge regarding new technologies and long-term working habits. Younger professionals reported being more prone to using digital tools than senior ones.

“Because I always have to look at a lot of things on the computer, uh, I'm not an individual who has much experience in using computers and it bothers me a bit to be watching, having to look at the screen” (Interviewee 6, male, 64 years old, 30 years of clinical practice, with mental health training, prescribing at an UCSP primary health care unit, used the ePrimaPrescribe DBCI platform).

“I might prefer things online, because I can access it more easily” (Interviewee 8, male, 31 years old, 6 years of clinical practice, with mental health training, prescribing at an USF primary health care unit, used the ePrimaPrescribe DBCI platform).

#### Digital infrastructure

Technical issues, such as bad internet connection and lack of sound devices were mentioned as significantly influencing implementation of our DBCI platform (Table 2). The lack of proper hardware at primary health care units to use digital platforms (proper internet connection, or computers’ configuration) was pointed to as an important barrier, even when the GP had a high motivation to comply with CME. This was exemplified by a case where the GP chose to print the content of the DBCI because he could not access the platform during office hours.

“I tried to get into USF X at the time…and I couldn't…and then there was no sound and then I couldn't load…and then it was blocked…and therefore, the accesses I ended up doing were at home… and then when I needed it at the I work, to clarify any doubts… I couldn't use it” (Interviewee 8, male, 31 years old, 6 years of clinical practice, with mental health training, prescribing at an USF primary health care unit, used the ePrimaPrescribe DBCI platform).

#### Motivational and emotional factors

In general GPs reported low motivation to comply with CME, especially after their junior residency period. Senior GPs mentioned that residents (hence younger and during their compulsory residency training period) usually make an effort to comply with training but felt that they themselves had a lower motivation level to participate, mostly due to habit and the perception of a higher work overload. These were some of the reasons pointed out by GPs to excuse a certain therapeutic inertia, particularly concerning BZD prescription and starting BZD withdrawal schemes.

*"I understand that older or less patient colleagues no longer have the…motivation to do this type of training" (Interviewee 8, male, 31 years old, 6 years of clinical practice, with mental health training, prescribing at an USF primary health care unit, used the ePrimaPrescribe DBCI platform)‬‬‬‬‬‬‬‬‬‬‬‬‬‬‬‬‬‬‬‬‬‬‬‬‬‬‬‬‬‬‬‬‬‬‬‬‬‬‬‬‬‬‬‬‬‬‬‬‬‬‬‬‬‬‬‬‬‬‬‬‬‬‬‬‬‬‬‬‬‬‬‬‬‬‬‬‬‬‬‬‬‬‬‬‬‬‬‬‬‬‬‬‬‬‬‬‬‬‬‬‬‬‬‬‬‬‬‬‬‬‬‬‬‬‬‬‬*

*“I don't have much patience anymore because I'm going to retire after year!” (Interviewee 10, female, 64 years old, 38 years of clinical practice, without mental health training, prescribing at an USF primary health care unit, did not use the ePrimaPrescribe DBCI platform)‬‬*

The influential factors regarding motivation ranged from problems with changing old routines (Table 2) and therapeutic inertia, to a lack of self-discipline and laziness. GPs related these factors to their difficulty in setting limits between work and leisure time, once again stressing the issue of time as limiting compliance to CME. Some participants stated that a lack of interest in the specific theme of an offered CME intervention would negatively determine their compliance. The general lack of motivation led to feelings of guilt and overwhelm, with the latter being a frequently mentioned argument for non-compliance with CME.

"Afterwards, at home, few colleagues have the will or patience to go open another platform and see something else… they were and are too fed up with work to go do or think about work when they get home" (Interviewee 8, male, 31 years old, 6 years of clinical practice, with mental health training, prescribing at an USF primary health care unit, used the ePrimaPrescribe DBCI platform).

"At a certain moment I realized that I was late… and, and that also made me feel a little guilty… deep down I felt that I was failing" (Interviewee 7, male, 60 years old, 33 years of clinical practice, with mental health training, prescribing at an UCSP primary health care unit, did not use the ePrimaPrescribe DBCI platform).

### Facilitators

#### Convenience of delivery method

GPs considered that one of the main facilitators of CME implementation and successful compliance would be the convenience of its delivery method (Table 2). However, when specifying what method would be most convenient, the perspectives seemed antagonistic. Most participants mentioned that they would prefer onsite CME, arguing that they felt that they learned better in such a setting, since it was easier to stay concentrated and avoid distractions.

"The ideal would be things in person, reduced audience, and maybe with a little more frequency. That is, I don't know, every three months, every two months, talk about it…or remember, or give… training, or even if it was just once or twice a year, but with tools that you could use. I think it would be, it would be what would work out better" (Interviewee 8, male, 31 years old, 6 years of clinical practice, with mental health training, prescribing at an USF primary health care unit, used the ePrimaPrescribe DBCI platform).

It was also said that onsite training, such as conferences or periodic meetings with experts, allowed GPs to escape their routine, and made it easier to establish limits between work and off-work time. Junior and senior GPs shared the preference for onsite training, although the reasons presented for this preference were different. Senior doctors mentioned preferring onsite training due to technical difficulties and personal preference; junior doctors seemed to prefer onsite training as a way of assuring the protection of their time to comply with CME.

“For me, it would always be more advantageous one, one, one training, even if it was one day a week… or two days a week that I could manage… and at that time I'm just for this… for me it's more profitable than, trying to read, because, and we would be talking to each other…” (Interviewee 7, male, 60 years old, 33 years of clinical practice, with mental health training, prescribing at an UCSP primary health care unit, did not use the ePrimaPrescribe DBCI platform).

For senior GPs onsite CME was also preferred to online due to their lack of openness to digital tools.

"I prefer one with, training with the person present…no, things on the internet, no, don't invite me" (Interviewee 10, female, 64 years old, 38 years of clinical practice, without mental health training, prescribing at an USF primary health care unit, did not use the ePrimaPrescribe DBCI platform).

Some GPs claimed advantages of an online delivery method, arguing for the feasibility of implementing CME online platforms implementation, for the ease of access to knowledge and the ease of access concerning technical aspects, such as the possibility of using it at any time according to personal availability. For these GPs, CME available online is more convenient, avoids unnecessary travel, responds to the need to be updated, and is an accessible way to improve one’s knowledge and practice. The only downside they acknowledged relating to this delivery method was, once again, to time management, since most GPs claimed they were not supposed to use their 15 days’ leave a year on this type of CME. Notwithstanding, even for GPs advocating for online CME options, it was recognized it was helpful to have an onsite presentation of digital platforms.

“It's a good option, especially for these issues of time and travel. It can even be done anywhere, anywhere…" (Interviewee 11, male, 30 years old, 6 years of clinical practice, with mental health training, prescribing at an USF primary health care unit type, did not use the ePrimaPrescribe DBCI platform).

#### Practical and pragmatic content

Participant GPs suggested specific content characteristics to facilitate CME implementation. Most highlighted the specificity and didactic benefits (Table 2), such as focusing on prescribing and deprescribing processes and on specific pathologies. Regarding online CME, they focused on the need for a nice interface and a simple structure.

"I think it would have to be more, uh, more practical, let's say, it would have to be a more practical training. I'm not saying that no to the issue of pharmacology and all of this, but, uh, [it should] be a thing, uh, eminently practical, with, comprehensive in relation to the various types of treatments that exist, therapies that exist, but it would be more, more practical" (Interviewee 4, male, 63 years old, 37 years of clinical practice, without mental health training, prescribing at an UCSP primary health care unit, used the ePrimaPrescribe DBCI platform).

#### Liaison with specialists

### Mandatory CME

“Theoretically there should be a part of our working time to, for personal training, hmm, to read articles, including distance training, which nowadays is often more practical, hmm, and more economical, hmm, in practice. Speaking for myself, I spend, um, ninety-nine percent of my professional time seeing patients" (Interviewee 7, male, 60 years old, 33 years of clinical practice, with mental health training, prescribing at an UCSP primary health care unit type, did not use the ePrimaPrescribe DBCI platform).

“I don't know, if this comment fits here. I think that, in our training, over the years, maybe there should be mandatory training, right? …if we were somehow obligated, in quotes, I don’t mean doing training all the time, but if there were some things that were mandatory, I'm sure people would go there, would do it, whether through… of platforms, whether it was otherwise, isn't it?” (Interviewee 3, female, 64 years old, 38 years of clinical practice, without mental health training, prescribing at an USF primary health care unit, used the ePrimaPrescribe DBCI platform).

Taking into account all the factors mentioned in the examples above, the barriers to CME identified by GPs are manifold, and reveal the entanglement of organizational and personal, or more subjective, factors in the perception of GPs about CME in its different possible modalities. The table below synthesizes these results, of the BaFAI questionnaire, and the globality of factors considered by GPs as barriers to CME:

## Discussion

The primary context framing our narratives were GPs’ perspectives on the implementation process of a DBCI, after being involved in an effectiveness cluster-randomized trial aiming to change their prescription patterns. The reported content, derived from two different questionnaires and in-depth interviews, was enlarged to discussing CME in the primary health care settings and the possibility of delivering CME through online platforms such as our DBCI.

### Impact, effect size and motivation to participate

When questioned about their motivations to use and expectations coming from using our DBCI platform, or more generally towards CME compliance, knowledge acquisition and practice improvement were frequently mentioned. Research by Kelly reports similar findings regarding motivation, stating that doctors felt that gaining knowledge was a good reason to comply with CME [20]. A meta-analysis regarding CME effectiveness also suggests that the effect size of CME on physician knowledge is medium, though the effect size is small for physician performance and patient outcomes [3]. Thus, there seems to exist a discrepancy between the expected and the real impact of CME implementation. This is in agreement with our effectiveness trial, since after the implementation of the intervention we could not find any significant change in prescription patterns. The reasons for this might be related to a CME being a life-long process, with behaviour change being an incremental, evolutionary process, with reinforcement of knowledge from different sources, and where single events are rarely perceived to effect change [21]. Also, we recognise that a limited-time program without the interaction and case discussion between participants that happen for instance in academic detailing [22], would predictably have less impact than more longitudinal, multimodal educational efforts with multiple exposures [3, 23].

### Barriers

Time was consistently pointed out, both by the interviewed GPs in this study and by a large body of literature, as the main barrier to CME implementation [19, 21, 24–28]. Time has been reported to transversally affect CME implementation, negatively influencing GPs motivation, satisfaction, and availability, and even their self-perceived role, leading to frustration and guilt [29], and regardless of age or clinical experience. Time management was considered to be even more challenging with online CME, since participants mentioned only being able to take part in this type of intervention in their free time. In some ways, time appears to be a quick and easy justification, pointing to the need for a profound reflection on the possible ways to change this challenging aspect of primary health care settings to allow for the integration of regular and effective CME.

GPs feel, in general, that their training is overall insufficient to handle the variety of clinical situations they have to solve, given that they are in the first line of medical care. Consequently, that they acknowledge that they need to receive continuous training in many different areas. This perception, when confronted with the lack of time, leads again to negative feelings of a lack of discipline, laziness, and of guilt, as well as saturation with work. Indeed, the assumptions that GPs have a manageable workload appears questionable at a time when there is a worldwide strategic shift of responsibility for health onto primary care [30].

GPs often mentioned emotional barriers limiting CME implementation, relating to their perceptions of work and bureaucracy overload, saturation from daily work, and guilt. This points to a general underlying negative perspective and sense of low satisfaction towards their daily clinical role and performance. We might consider this as inevitably jeopardizing any motivation to attend CME, regardless of the type and content of the training. A recent study discussed GPs’ wellbeing as an important factor regarding quality of care, GP prescribing patterns, patients’ adherence to medical treatments and patient satisfaction [31]. Other evidence discusses reasons for a general feeling of unhappiness and dissatisfaction, reporting that when doctors gather, their conversation turns to misery and talk of early retirement. This suggests that the cause for this dissatisfaction, which in some ways is also found in our data, might be a breakdown in the implicit compact between doctors and society: the individual orientation that doctors are trained for does not fit with the demands of current healthcare systems [32, 33].

Older age and greater clinical experience were frequently cited as having a negative influence on GPs’ availability for training, suggesting that the opposite—younger age and lower number of years of clinical experience—would be associated with higher levels of motivation to attend CME. However, this might be only true for GPs during their residency/formal training period, since the narratives of young GPs revealed a similar discourse as the ones of senior doctors, pointing to time limitations as a significant barrier to CME. The existing literature regarding age and clinical experience is in accordance with our findings [34], pointing to other factors, such as time and perception of work overload, as more significantly and transversally influencing doctors’ compliance with CME [26].

Lack of digital savvy and lack of proper hardware at primary health care units were also mentioned as important barriers to the implementation and acceptance of online CME, especially for senior GPs. This finding is supported by further evidence, reporting that senior GPs with more years in clinical practice are less likely to have access to or to use computer-based technologies [27, 34, 35]. However, this might be a changing reality. The current global COVID-19 pandemic situation has, for example, forced many senior doctors who previously refused, due to habit or inertia, to use new technology to adapt and integrate online communication in their clinical practice. Indeed, the idea of the need for a “forced or mandatory” change, taking place within working hours, as a rapid way to lead to desired/effective outcomes was reflected both in our participants’ perceptions and in the literature [36, 37]. Participants reported that acceptance of online training would improve if preceded and periodically accompanied by onsite training, onsite practical discussions with specialists or, at least, an initial onsite presentation of online materials.

### Facilitators

Considering the convenience of the CME delivery method regarding to acceptability, and the opposing perspectives regarding onsite versus online preferences, the literature indicates a general preference for live training due to the value of personal interaction and the perception that CME that does not involve personal interaction was adjunctive [24, 28, 35]. However, it has to be recognized that onsite CME may, in many cases, represent a cost-ineffective option, due to the also very limited, availability of specialists. This is a rather interesting future research subject to be pursued after the COVID-19 pandemic, since it the pandemic has been a period where an exponential increase in the use of new technologies to support clinical practice took place.

Having easy access to some kind of interaction with specialists allows for clarification, the personalization of information, exploration, feedback, and reflection. It can also address other needs of doctors that may not be recognized or quantified, such as the need for support, recognition, motivation and fulfilment, and the ‘need’ to belong to a professional community. Thus, it would also be interesting to explore in future research the suggestion of complementing innovative CME delivery methods with a facilitated way of liaising with specialists, such as the implementation of a telephone helpline, as a way of guaranteeing a middle-ground option between onsite and online CME.

Another key solution pointed out by participants was for CME to be mandatory. In fact, “forcing the change” is a significant aspect of our narratives, translated by a common perspective that CME implementation would only be possible through the implementation of obligatory training [36]. Participant GPs pointed to this as the only way to protect training time after residency, which is also in accordance with other countries’ experience [37]. However, there is no clear and compelling body of research that demonstrates that mandatory CME ultimately results in improved GP learning [21]. In countries where mandatory CME has been implemented, it has been argued that it devalues and discourages other forms of learning, and that it can detract from a focus on the identification of current or pressing educational needs, the addressing of those needs and the development of reflections about the learning process [21]. Notwithstanding, and as another argument for mandatory training, it has to be recognized that CME cannot be entirely focused on GP preferences [28]. If implemented in within a reflexive framework, that would allow for participatory decision making about training innovations, novel subjects and formats, mandatory training would push GPs to engage with medical subjects that otherwise they would otherwise probably neglect, while simultaneously protecting training hours within work schedules.

## Conclusion

This study is, to our knowledge, the first to explore the barriers and facilitators perceived by GPs in Portugal to CME implementation. As mentioned, primary health care in Portugal is the main branch of health care responsible for the provision of care and has a gatekeeping function for hospital care. In this sense, the results of this study can be applied to countries where a similar NHS exists, and where primary health care plays a central role in the provision of care.

We recognise as the main limitation to our study the small sample size of participant GPs, especially regarding qualitative data coming from the in-depth interviews. Notwithstanding, our findings are generally consistent with recent literature regarding physician perceptions and preferences for type of CME/professional development activities, and use of educational technologies (including online platforms) for professional development, and barriers to implementing learning from CME. More specifically, and in agreement with our data, a modest desire for more online learning is reported [34], and the perception of the effectiveness of, access to, and future role of educational technologies has been noted to vary minimally across age groups [34] in other locations. Additionally, further research reported time, patient, organizational, and provider factors, as the main barriers to implementing CME, which is also consistent with our findings [19, 38]. When considering time as the main consensual barrier to CME implementation, and despite the fact that online training is often touted as convenient by its on-demand nature, it should be highlighted how time management was referred to be challenged by online CME, due to the promiscuity between work and free time it demanded, itself related to further infrastructural factors. This was a major barrier, indicative of broader career dissatisfaction.

We consider one of the main strengths of our research to be the choice of a mixed-methods approach, which combined different data sources, allowing for an in-depth acknowledgement of the factors involved in CME implementation. One of the data sources, the BaFAI, also documented that the barriers and facilitators to implementing the DBCI platform used for our effectiveness cluster randomized trial were similar to barriers and facilitators found in our control group, which used a similar methodology and format but a different content. This suggests that, when considering the implementation of a DBCI aimed at changing GPs’ behaviour in primary health care settings, our reported barriers—the need for time investment; the general attitudes towards the DBCI; the motivation and self-perceived role of the professionals involved; and the resistance to a change in old practice habits—are worthy of inspection and consideration, regardless of the content of the intervention. The results also show, clearly, that a DBCI is difficult to implement if adequate instruments and facilities are not available. Furthermore, and in a broader sense, the implementation of CME requires deep reflection integrating bottom-up perspectives, since successful implementation will always significantly depend on perceived challenges coming from daily routine clinical practice.

## Supplementary Information

**Additional file 1.**

## Data Availability

The datasets used and/or analysed during the current study are available from the corresponding author on reasonable request. The results of this study were disseminated via peer-reviewed publications and conference presentations. All data will be available on request.

## Abbreviations

BaFAI: Barriers and Facilitators Assessment Instrument
BZD(s): Benzodiazepine(s)
CME: Continuing medical education
DBCI: Digital Behaviour Change Intervention
GP(s): General Practitioner(s)
NHS: National Health Service
UCSP: Personalized care unit model (type of organizational model for primary health care units in Portugal)
USF: Family health unit model (type of organizational model for primary health care units in Portugal)
