# Supplementary material for: Barriers and facilitators to implementing a continuing medical education intervention in a primary health care setting
Source: BMC Health Serv Res. 2022 May 13;22:638. doi: 10.1186/s12913-022-08019-w (PMC9099036; doi:10.1186/s12913-022-08019-w)
Supplement: Supplementary file 1 — Additional file 1. [file 12913_2022_8019_MOESM1_ESM.docx]

**Additional Files**

Additional file 1: 6-question onsite questionnaire

| Question | Type of question |
| --- | --- |
| Degree of motivation to participate in this study | Multiple answer question with the following answer options:  a) None  b) Little  c) Some  d) A lot |
| Do you think it is feasible to implement online training programs like ePrimaPrescribe? | Dichotomic answer question with the following answer options:  a) yes  b) no |
| Do you consider that the use of the ePrimaPrescribe platform will have an impact on changing your benzodiazepine prescription pattern? | Dichotomic answer question with the following answer options:  a) yes  b) no |
| State your main motivations for using the ePrimaPrescribe platform | Short answer question |
| State the main barriers you expect to encounter to using the ePrimaPrescribe platform. | Short answer question |

Additional file 2: Characteristics of onsite questionnaire responders

|  | Used ePrimaPrescribe online platform | | Didn’t use ePrimaPrescribe online platform | |
| --- | --- | --- | --- | --- |
|  | USF | UCSP | USF | UCSP |
| Female  n (%);  Male  n (%) | 15 (62,5%)  4 (30,8%) | 3 (12,5%)  3 (12,5%) | 3 (12,5%)  5 (38,5%) | 3 (12,5%)  1 (7,7%) |
| Age  n,  mean,  sd, (min,max) | 19  49,7  13  (30,67) | 6  56,5  10,1  (39,65) | 8  47,4  16,54  (28,66) | 4  55,8  8,66  (44,64) |
| Years of clinical experience, n,  mean,  sd, (min,max) | 19  23,1  12,48  (3,40) | 6  27,83  11,36  (8,40) | 8  20,75  15,8  (3,39) | 4  29,75  8,1  (19,38) |
| Training in mental health  Yes  No | 10 (47,6%)  9 (56,3%) | 4 (19%)  2 (12,5%) | 5 (23,8%)  3 (18,8%) | 2 (9,5%)  2 (12,5) |

Additional file 3: Characteristics of BaFAI responders

|  | Control | | Intervention | |
| --- | --- | --- | --- | --- |
|  | USF | UCSP | USF | UCSP |
| Female  n (%);  Male  n (%) | 6 (46%)  7 (54%) | 0 (0%)  4 (100%) | 16 (89%)  2 (11%) | 3 (50%)  3 (50%) |
| Age  n,  mean,  sd, (min,max) | 13  46.8  11.75  (34,63) | 4  54.5  12.71  (43,76) | 18  50.22  11.57  (32,67) | 6  56,5  10.07  (39,65) |
| Years of clinical experience, n,  mean,  sd, (min,max) | 13  19.15  12.18  (7,38) | 4  28.5  11.56  (18,39) | 18  23.5  10.93  (5,40) | 6  27.83  11.35  (8,40) |
| Training in mental health  Yes  No | 8 (62%)  5 (38%) | 2 (50%)  2 (50%) | 7 (39%)  11 (61%) | 4 (67%)  2 (33%) |

Additional file 4: Characteristics of interview responders

| Characteristics of interview responders | n (% of total) |
| --- | --- |
| Type of primary health care unit  USF  UCSP | 7 (58)  5 (42) |
| Specific training in mental health  Yes  No | 6 (50)  6 (50) |
| Gender  Female  Male | 5 (42)  7(58) |
| Usage of ePrimaPrescribe  Yes  No | 7 (58)  5 (42) |
| Age | Mean: 54.25  SD: 14.88  (Min,Max) (30,67) |
| Years of clinical practice | Mean: 26.8  SD: 14.56  (Min,Max) (5,40) |
